# Prevalence of autistic traits and their relationships with other psychopathological domains in young adults seeking psychiatric attention: a cluster analysis

**DOI:** 10.1192/j.eurpsy.2024.1791

**Published:** 2024-10-23

**Authors:** Benedetta Demartini, Gianmarco Ingrosso, Francesca Serio, Veronica Nisticò, Giovanni Broglia, Angelo Bertani, Raffaella Faggioli, Orsola Gambini, Gabriele Massimetti, Liliana Dell’Osso, Barbara Carpita

**Affiliations:** 1 Unità di Psichiatria 51-52, San Paolo Hospital, ASST Santi Paolo e Carlo, Milan, Italy; 2 “Aldo Ravelli” Research Centre for Neurotechnology and Experimental Brain Therapeutics, University of Milan, Milan, Italy; 3 Department of Health Sciences, University of Milan, Milan, Italy; 4 Department of Psychology, University of Milano-Bicocca, Milan, Italy; 5 Centro Giovani “Ettore Ponti”, Mental Health and Dependences Department, ASST Santi Paolo e Carlo, Milan, Italy; 6 Department of Clinical and Experimental Medicine, University of Pisa, Via Roma 67, Pisa 56127, Italy

**Keywords:** Autism Spectrum Disorders, Dimensional Psychopathology, Empathy, Sensory Sensitivity, Affective Disorders, Anxiety, Depression, Eating Disorders, Personality disorders, Psychotic Disorders, Youth Mental Health

## Abstract

**Background:**

Nearly two-thirds of individuals with a mental disorder start experiencing symptoms during adolescence or early adulthood, and the onset of a mental disorder during this critical life stage strongly predicts adverse socioeconomic and health outcomes. Subthreshold manifestations of autism spectrum disorders (ASDs), also called autistic traits (ATs), are known to be associated with a higher vulnerability to the development of other psychiatric disorders. This study aimed to assess the presence of ATs in a population of young adults seeking specialist assistance and to evaluate the study population across various psychopathological domains in order to determine their links with ATs.

**Methods:**

We recruited a sample of 263 adolescents and young adults referring to a specialized outpatient clinic, and we administered them several self-report questionnaires for the evaluation of various psychopathological domains. We conducted a cluster analysis based on the prevalence of ATs, empathy, and sensory sensitivity scores.

**Results:**

The cluster analysis identified three distinct groups in the sample: an AT cluster (22.43%), an intermediate cluster (45.25%), and a no-AT cluster (32.32%). Moreover, subjects with higher ATs exhibited greater symptomatology across multiple domains, including mood, anxiety, eating disorder severity, psychotic symptoms, and personality traits such as detachment and vulnerable narcissism.

**Conclusions:**

This study highlights the importance of identifying ATs in young individuals struggling with mental health concerns. Additionally, our findings underscore the necessity of adopting a dimensional approach to psychopathology to better understand the complex interplay of symptoms and facilitate tailored interventions.

## Introduction

Despite substantial progress to date, mental illness continues to impose a significant burden on young individuals worldwide [1]. While most of the research has focused on the management of full-blown psychiatric syndromes in adult patients, early intervention and prevention have received comparatively less attention [2, 3]. Notably, nearly two-thirds of individuals with a mental disorder (62.5%) experience symptoms during adolescence or early adulthood (before the age of 25), with a median onset age of 18 [4]. These life phases are pivotal for establishing the sociocultural and emotional foundations for the transition into adulthood, and prospective analyses indicate that the onset of a mental disorder during this critical life stage strongly predicts adverse socioeconomic and health outcomes [5].

From a neuropsychological point of view, research has also highlighted the concept of “sensitive periods,” that is, phases in which risk factors can influence the manifestation of mental health symptoms, thereby providing potential timeframes for early intervention [3]. These considerations underscore the need to effectively address the mental health needs of young individuals, recognizing youth mental health as a distinct sector and prompting the development of services dedicated to early and preventive interventions [1].

### Autistic traits and a dimensional approach to psychopathology

Autism spectrum disorders (ASDs) are neurodevelopmental conditions characterized by persistent deficits in social communication and interactions, restricted and repetitive interests and behaviors, and altered sensory sensitivity. ASDs often remain unrecognized until adulthood, coming to clinical attention after the onset of other mental disorders, often prompted by life stressors [6, 7]. It is now recognized that autism features extend beyond those with a clinical diagnosis, existing on a continuum within the general population and manifesting at milder levels as subthreshold autistic characteristics [8, 9]. There is also evidence that, even when subthreshold, autistic traits (ATs) may be associated with a higher vulnerability to the development of other psychiatric disorders [10–14].

Current psychopathological research has pointed out the shortcomings of a categorical approach to mental illnesses, emphasizing the limitations of the DSM-5 concept of “comorbidity.” This model is more suitable when there is homogeneity between members of a class, clear boundaries among different classes, and when the classes are mutually exclusive, circumstances that are rarely encountered in clinical practice. On the other hand, a dimensional approach to psychopathology allows the description of patients across multiple syndrome dimensions that, in turn, constitute broad spectra of interrelated psychopathologies [15]. This approach is even more paramount in consideration of subthreshold symptomatic expression of psychopathology and calls for a novel diagnostic approach [3].

In light of the aforementioned considerations, this study aimed to characterize the presence of ATs, using a cluster analysis, in a population of young adults seeking specialist assistance. Alongside, the study sought to assess the study population across various psychopathological domains (mood, anxiety, feeding and eating disorders [EDs], symptoms of the psychotic sphere, and personality structure), in order to evaluate their links with ATs.

## Methods

### Study population

The study included a sample of 263 adolescents and young adults aged 16 to 24 attending the “Centro Giovani Ettore Ponti” (ASST Santi Paolo *e* Carlo, Milan, Italy), an outpatient clinic open to individuals who are experiencing distress, discomfort, or psychopathological suffering, whether at the onset or already manifested in behaviors or symptomatic expressions of any kind.

Study participants underwent an evaluation by a psychiatrist and a psychologist according to DSM-5-TR criteria [8]. Specifically, the Structured Clinical Interview for DSM-5 Disorders: Clinical Version (SCID-5-CV) [16] was administered for the diagnosis of psychiatric disorders. The SCID-5-SPQ [17] and the SCID-5-PD [18] were used, respectively, for screening and diagnosis of personality disorders. Informed written consent was obtained from every participant after a complete description of the study and with the opportunity to ask questions. Subjects were not paid for their participation and were free to withdraw from the study at any time without giving further explanation.

The study was conducted in accordance with the Declaration of Helsinki. The Ethics Committee of the ASST Santi Paolo *e* Carlo of Milan approved all recruitment and assessment procedures.

### Psychometric instruments

In order to collect specific information for the study, the following self-report questionnaires were administered: (i) the *Autism Quotient* [19], designed to measure the extent to which an adult without intellectual disabilities exhibits ATs; (ii) the *Ritvo Autism and Asperger Diagnostic Scale-Revised*, RAADS-R [20], usually implemented in clinical settings to support the diagnosis of ASDs without intellectual disabilities; (iii) the *Empathy Quotient*, EQ [21], to assess the level of cognitive and affective empathy; (iv) the *Sensory Perception Quotient – Short Form*, SPQ-SF35 [22], which investigates hypersensitivity or hyposensitivity across the five sensory modalities (sight, hearing, smell, touch, and taste) and has demonstrated good discriminatory ability between adults with ASDs and neurotypical individuals; (v) the *Beck Depression Inventory*, BDI-II [23], for the evaluation of depressive symptoms; (vi) the *State–Trait Anxiety Inventory* [24], for measuring state anxiety, which is experienced in response to a situation (STAI-Y1), and trait anxiety (STAI-Y2), which is a part of personality; (vii) the *Eating Attitude Test-26 items*, EAT-26 [25, 26], for the assessment of the symptoms and specific concerns of EDs; (viii) the *Prodromal Questionnaire–short version*, PQ-16 [27], for the identification of prodromal symptoms of psychosis; (ix) the *Personality Inventory for DSM-5*, PID-5-BF [28], used to evaluate the personality structure according to the alternative dimensional model of the DSM-5; (x) the *Pathological Narcissism Inventory*, PNI [29, 30], which investigates the levels of narcissistic grandiosity and vulnerability. For each of the abovementioned scales, the validated Italian version was administered to the patients.

### Statistical analyses

Descriptive statistics were calculated for all sociodemographic, clinical, and psychometric variables; in particular, we analyzed the frequencies of patients scoring above the cutoff on screening questionnaires. Statistical significance was defined at α < 0.05, and all tests performed were two-tailed. A K-means cluster analysis based on the standardized AQ, RAADS, EQ, and SPQ total scores was performed in order to evaluate the specific distribution of autism spectrum-related measures in distinct but homogeneous groups. We used squared Euclidean distance for the divergence measure between cases. The method of iterative updating of clustered centroids was chosen for classifying cases, with the new clusters’ centers being calculated after all cases are assigned to a given cluster. The solution for K = 3 (i.e., three clusters) was the most satisfactory with small within-cluster variability compared to the differences between the centroids of the clusters. Subsequently, we performed chi-square tests in order to compare categorical variables (gender, scores above or below BDI-II, STAI, EAT-26, and PQ-16 cutoffs) among clusters. In the case of gender and the presence/absence of a score above the cutoff at STAI-Y2, Fisher’s exact p-value was also reported as some cells showed an expected count lower than 5.

The Kruskal–Wallis test was used for comparing continuous variables (BDI-II, STAI, EAT-26, PQ-16, PID-5-BF, and PNI scores).

Finally, a discriminant analysis was carried out on the three cluster groups in order to confirm the results of the cluster analysis and to identify the weights of BDI-II, STAI, EAT-26, PNI, PID-5-BF, and PQ-16 scores in discriminating groups.

All statistical analyses were performed using the Statistical Package for Social Science, version 29.0 (SPSS Inc.).

## Results

The mean age of our sample was 19.57 years (± 2.02). Most of the participants (158, 60.08%) identified themselves with the female gender, 88 subjects (33.46%) identified with the male gender, 12 (4.56%) declared themselves nonbinary, and 5 (1.90%) preferred not to disclose their gender. The average BMI was 22.60 kg/h2 (± 5.15): in particular, 49 (18.63%) participants were underweight (i.e., BMI below 18.5 kg/h2), 152 (57.78%) had a healthy weight (18.5 < BMI < 25 kg/h2), 41 (15.60%) participants were overweight (25 < BMI < 30 kg/h2), and 21 (7.99%) participants were obese (BMI above 30 kg/h2). Further sociodemographic information is reported in Table 1.

**Table 1.** Sociodemographic information

		Value
Age, mean ± SD		19.57 ± 2.02
BMI, mean ± SD		22.6 ± 5.15
BMI, N (%)	Underweight	49 (18.63)
	Healthy weight	152 (57.78)
	Overweight	41 (15.6)
	Obese	21 (7.99)
Sex assigned at birth (M/F)		90/173
Self-reported gender, N (%)	Female	158 (60.08)
	Male	88 (33.46)
	Nonbinary	12 (4.56)
	Preferred not to declare	5 (1.9)
Sexual orientation, N (%)	Asexual	8 (3.04)
	Bisexual	39 (14.83)
	Heterosexual	154 (58.56)
	Homosexual	9 (3.42)
	Pansexual	16 (6.08)
	Other	8 (3.04)
	Preferred not to declare	29 (11.03)
Education level, N (%)	Middle school license	93 (35.36)
	Professional high school license	19 (7.22)
	High school diploma	141 (53.61)
	Bachelor degree	9 (3.42)
	Master’s degree	1 (0.38)
Employment, N (%)	Student	197 (74.9)
	Employed	34 (12.93)
	Unemployed	32 (12.17)
Living environment, N (%)	Alone	5 (1.9)
	With family of origin	236 (89.73)
	With partner	5 (1.9)
	With flatmates	15 (5.7)
	Recovery community	2 (0.76)

Abbreviations: BMI, body mass index; F, female; M, male; N, numerosity; SD, standard deviation; %, percentage.

**Figure 1.**
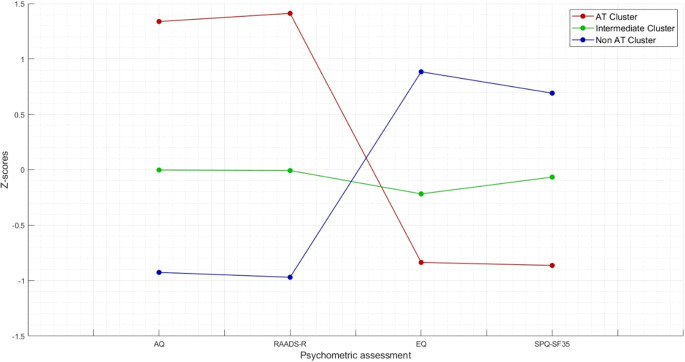
Z-score values of AQ, RAADS, EQ, and SPQ total scores in the three clusters.

The K-means cluster analysis met criterion 0 of convergence at the tenth iteration. The three clusters of subjects determined by the K-means cluster analysis were defined as AT cluster (n = 59, 22.43%), intermediate cluster (n = 119, 45.25%), and no-AT cluster (n = 85, 32.32%). Figure 1 shows the z-score values of AQ, RAADS, EQ, and SPQ total scores in the three clusters. The distance between AT and no-AT cluster centers was 4.021, the distance between AT and intermediate cluster centers was 2.196, and the distance between no-AT and intermediate cluster centers was 1.889. The average distance of cases from their cluster center was 1.239 ± 0.515. The dispersion analysis showed that AQ and RAADS scores were those with the greatest influence in forming clusters (F = 277.872 and F = 397.771, respectively) (Table 2).

**Table 2.** Dispersion analysis for cluster analysis based on AQ, RAADS-r, EQ, and SPQ-SF35 total scores

	Cluster mean square Z-scores (SE)	F	P
AQ total score	89.247 (0.321)	277.872	< 0.001
RAADS-R total score	98.732 (0.248)	397.771	< 0.001
EQ total score	56.659 (0.572)	99.079	< 0.001
SPQ-SF35 total score	42.543 (0.680)	62.523	< 0.001

Abbreviations: AQ, autism quotient; EQ, empathy quotient; RAADS-R, Ritvo Autism and Asperger Diagnostic Scale-Revised; SE, standard error; SPQ-SF35, Sensory Perception Quotient Short Form.

A significant difference in gender distribution emerged for the clusters (chi-square = 32.012; p < .001, Fisher’s exact p < .001). Females were significantly more represented in the no-AT group (n = 65; 76.5%) than in AT (n = 31; 54.4%) and intermediate ones (n = 62; 53.4%). Males were significantly more represented in the intermediate group (n = 52; 44.8%) than in the no-AT one (n = 19; 22.4%), while an in-between proportion was reported in the AT group (n = 17; 29.8%). Nonbinary subjects were significantly more represented in the AT group (n = 9; 15.8%) than in the no-AT (n = 1; 1.2%) and intermediate (n = 2; 1.7%). Five subjects (2 in the AT group and 3 in the intermediate group) preferred to not disclose their gender.

According to the Kruskal–Wallis analysis, subjects in the no-AT cluster scored significantly lower than the AT cluster on BDI-II total scores (data available for 193 subjects), and STAI-Y2 (data available for 196 subjects) scores. Noticeably, for STAI-Y1, AT subjects also reported significantly higher scores than intermediate cluster participants, while for STAI-Y2, AT and intermediate cluster groups did not score significantly different, both reporting a significantly higher score than no-AT group (Table 3). No difference was reported in the proportion of subjects scoring above the BDI-II cutoff: AT group = 38 (90.5%), intermediate group = 72 (83.7%), and no-AT group = 49 (75.4%); chi-square = 4.195, p = .127. Similarly, no difference emerged in the proportion of subjects scoring above the STAI cutoffs, although a tendency toward significance is observable for STAI-Y2. STAI-Y1: AT group = 38 (92.7%), intermediate group = 74 (83.1%), and no-AT group = 52 (78.8%); chi-square = 3.607, p = .161. STAI-Y2: AT group = 40 (97.6%), intermediate group = 86 (96.6%), and no-AT group = 57 (87.7%); chi-square = 6.436, p = .049, Fisher’s exact p = .051.

**Table 3.** Comparison of BDI-II and STAI, EAT-26, PQ-16, PID-5-BF, and PNI scores among cluster groups

	Mean ± SD, mean rank	Mean ± SD, mean rank	Mean ± SD, mean rank	H	p	Post hoc p < 0.05	Numerosity
BDI-II total score	35.71 ± 14.17, 121.92	29.48 ± 15.02, 98.20	25.20 ± 16.32, 79.32	14.921	<0.001	AT>no-AT	AT = 42 Intermediate = 86 No-AT = 65
STAI-Y1 total score	58.63 ± 11.87, 124.56	52.47 ± 12.12, 96.44	49.01 ± 14.09, 85.08	12.475	0.002	AT>intermediate AT>no-AT	AT = 41 Intermediate = 89 No-AT = 66
STAI-Y2 total score	63.34 ± 9.85, 123.66	59.61 ± 10.10, 102.89	52.91 ± 13.90, 75.12	19.845	<0.001	AT>no-AT Intermediate>no-AT	AT = 41 Intermediate = 89 No-AT = 65
EAT–26 dieting	9.85 ± 8.20, 160.24	6.71 ± 8.02, 126.20	6.09 ± 7.61, 120.52	10.865	0.004	AT>intermediate AT>no-AT	AT = 59 Intermediate = 119 No-AT = 85
EAT–26 bulimia	3.93 ± 4.28, 160.52	2.35 ± 3.81, 126.85	1.94 ± 3.23, 119.41	12.807	0.002	AT>intermediate AT>no-AT	
EAT– 26 oral control	5.34 ± 4.51, 169.22	2.84 ± 3.47, 127.63	2.05 ± 2.62, 112,29	21.138	<0.001	AT>intermediate AT>no-AT	
EAT–26 total score	19.12 ± 13.67, 172.83	11.91 ± 13.31, 124.77	11.52 ± 1.25, 113.78	23.011	<0.001	AT>intermediate AT>no-AT	
PQ–16 symptoms	5.98 ± 3.54, 118.63	4.95 ± 2.98, 102.97	3.80 ± 2.63, 81.56	12.003	0.002	AT>no-AT	AT = 45 Intermediate = 88 No-AT = 65
PQ–16 distress	12.31 ± 9.85, 82.63	7.83 ± 5.14, 67.97	6.00 ± 4.32, 53.73	10.344	0.006	AT>no-AT	
PID–5-BF negative affect	9.61 ± 3.05, 112.44	8.20 ± 3.06, 88.40	7.44 ± 3.42, 79.06	9.708	0.008	AT>no-AT	AT = 37 Intermediate = 82 No-AT = 62
PID–5-BF detachment	8.47 ± 2.64,126.11	6.22 ± 3.27, 89.81	4.94 ± 2.69, 69.27	27.642	<0.001	AT>intermediate AT>no-AT	
PID–5-BF antagonism	3.53 ± 3.06, 92.10	3.65 ± 3.05, 94.49	3.13 ± 3.19, 82.92	1.855	0.396		
PID–5-BF disinhibition	7.19 ± 2.82, 118.22	4.84 ± 3.04, 80.81	5.35 ± 3.97, 85.61	13.794	0.001	AT>intermediate AT>no-AT	
PID–5-BF psychoticism	7.86 ± 3.64, 118.38	5.96 ± 3.17, 92.92	453 ± 3.43, 69,71	20.726	<0.001	AT>intermediate>no-AT	
PID–5-BF total score	37.78 ± 10.97, 129.12	28.70 ± 9.92, 87.88	25.39 ± 11.86, 72.38	27.738	<0.001	AT>intermediate AT>no-AT	
PNI contingent self-esteem	3.18 ± 1.07, 159.71	2.85 ± 1.02, 135.58	2.38 ± 1.20, 107.76	16.733	<0.001	AT>no-AT Intermediate>no-AT	AT = 59 Intermediate = 119 No-AT = 85
PNI exploitativeness	1.95 ± 0.85, 116.49	2.20 ± 0.90, 137.26	2.21 ± 0.89, 135.40	3.210	0.201		
PNI self-sacrificing self-enhancement	2.97 ± 0.97, 138.93	2.92 ± 0.96, 133.09	2.81 ± 0.93, 125.66	1.108	0.575		
PNI hiding the self	3.52 ± 0.99, 158.31	3.26 ± 0.87, 134.19	2.89 ± 1.12, 110.68	13.849	<0.001	AT>no-AT	
PNI grandiose fantasy	2.84 ± 1.28, 142.83	2.90 ± 1.12, 147.81	2.16 ± 1.15, 102.35	19.261	<0.001	AT>no-AT Intermediate>no-AT	
PNI devaluing	2.84 ± 1.07, 176.21	2.23 ± 1.04, 134.55	1.66 ± 1.06, 97.75	37.329	<0.001	AT>intermediate>no-AT	
PNI entitlement rage	2.64 ± 1.39, 147.27	2.56 ± 1.04, 140.44	2.90 ± 1.00, 109.59	11.238	0.004	AT>no-AT Intermediate>no-AT	
PNI narcissistic grandiosity	2.58 ± 0.83, 133.44	2.67 ± 0.80, 142.32	2.39 ± 0.72,116.55	5.717	0.057		
PNI narcissistic vulnerability	3.04 ± 0.91, 166.39	2.73 ± 0.80, 137.34	2.25 ± 0.90, 100.65	27.088	<0.001	AT>intermediate>no-AT	
PNI total score	2.84 ± 0.79,153.95	2.70 ± 0.72,141.43	2.32 ± 0.75, 103.56	18.619	<0.001	AT>no-AT Intermediate>no-AT	

Abbreviations: AT, autistic trait; BDI-II, Beck Depression Inventory – Second Edition; EAT-26, Eating Attitude Test-26 items; PID-5-BF, Personality Inventory for DSM-5-Brief From; PNI, Pathological Narcissism Inventory; PQ-16, Prodromal Questionnaire Short Version; STAI-Y1, State–Trait Anxiety Inventory – State Anxiety; STAI-Y2, State–Trait Anxiety Inventory – Trait Anxiety.

Subjects in the AT cluster scored significantly higher than the other two on EAT-26 total and domain scores (Table 3), while for PQ-16 (data available for 198 subjects) the only significant difference was reported between AT and no-AT clusters, the latter scoring significantly lower than the first at both symptom and distress scores (Table 3). Moreover, the AT group reported a significantly higher proportion of subjects scoring above the cutoff of EAT-26 when compared with the other two groups: AT group = 24 (40.7%), intermediate group = 23 (19.3%), and no-AT group = 15 (17.6%); chi-square = 12.428, p = .002. The AT group also reported a significantly higher proportion of subjects scoring above the cutoff of PQ-16, only when compared with the no-AT group: AT group = 18 (40.0%), intermediate group = 21 (23.6%), and no-AT group = 9 (13.8%); chi-square = 9.962, p = .007.

Considering the PID-5 BF scale (data available for 181 subjects), AT subjects scored significantly higher than the other two clusters on the total score and on *Detachment* and *Disinhibition* factors, while for the *Negative affect* factor the AT cluster scored only higher than the no-AT one. For *Psychoticism*, AT subjects scored significantly higher than the other two clusters, while, in turn, the intermediate cluster scored higher than the no-AT one. No significant difference was found for the *Antagonism* factor (Table 3).

Finally, on PNI, AT and intermediate cluster groups scored significantly higher than the no-AT group on total scores; no significant differences were reported for *Narcissistic grandiosity* factor and for the subscales *Exploitativeness, Self-sacrificing Self-enhancement*, while AT and intermediate clusters scored higher than no-AT on *Grandiose fantasy* and *Entitlement rage.* On the other hand, AT group scored significantly higher than the intermediate cluster on *Narcissistic vulnerability*, which in turn scored higher than the no-AT one. In all the three subscales of *Contingent Self-esteem, Hiding the self*, and *Devaluing*, AT subjects scored higher than no-AT ones, while the intermediate cluster scored higher than the no-AT only on *Contingent self-esteem* and *Devaluing* (Table 3).

**Figure 2.**
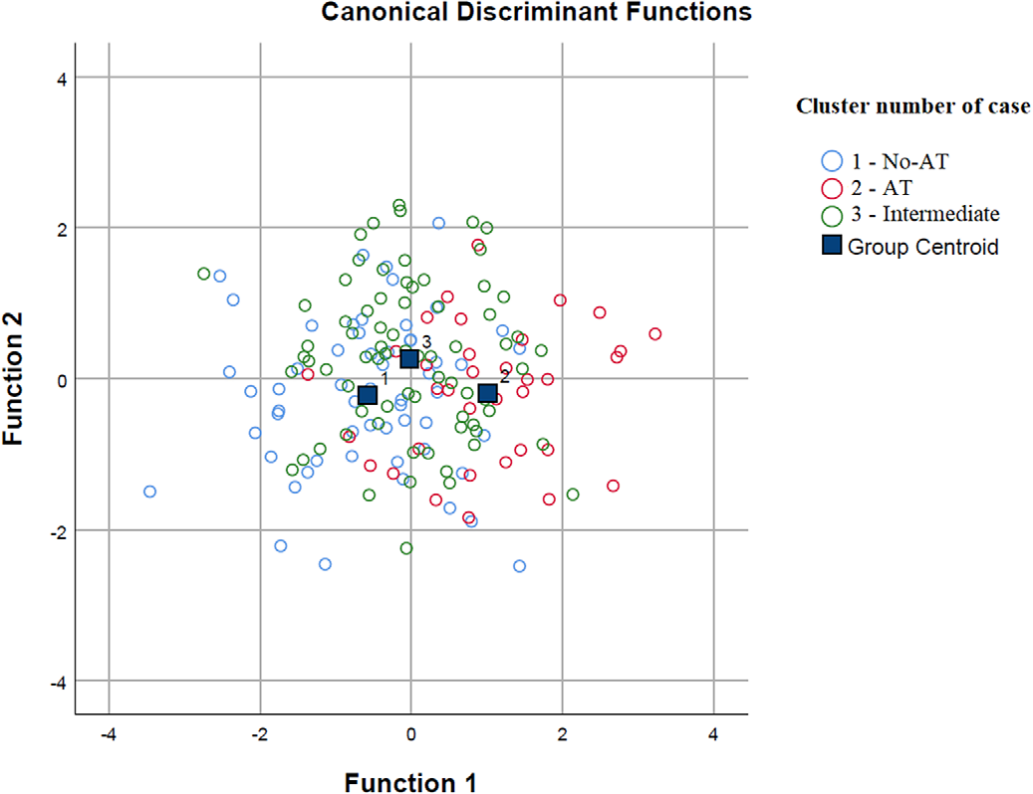
Discriminant analysis: group graphic.

According to the discrimination analysis, two functions were identified: function 1 adsorbed most of the variance (85.9%), while function 2 absorbed 14.1% of the variance (Table 4 and Figure 2). The discriminating elements in function 1, in order of higher discriminant value, were PID-5-BF total, STAI-Y2 total, EAT-26 total, STAI-Y1 total, PQ-16 symptoms total, BDI-II total, and PNI *Narcissistic vulnerability* total. Function 2 was represented only by the PNI *Narcissistic grandiosity* total (Table 5).

**Table 4.** Summary of canonical discriminant functions for discriminant analysis

Function	Eigenvalue	% of variance	Cumulative %	Canonical correlation
1	0.339	85.9	85.9	0.503
2	0.056	14.1	100	0.230
**Test of functions**	**Wilks’ lambda**	**Chi-square**	**Df**	**Sig**
1 through 2	0.708	54.809	16	0.000
2	0.947	8.584	7	0.284

**Table 5.** Structure matrix for discriminant analysis

	Function	
	1	2
PID–5-BF total score	0.743*	−0.198
STAI-Y2 total score	0.592*	0.333
EAT–26 total score	0.473*	0.103
STAI-Y1 total score	0.467*	−0.256
PQ–16 symptoms total score	0.436*	0.058
BDI-II total score	0.420*	0.034
PNI narcissistic vulnerability total score	0.412*	0.332
PNI narcissistic grandiosity total score	0.023	0.505*

* Largest absolute correlation between each variable and any discriminant function.

## Discussion

The characterization of the sample based on the prevalence of ATs, empathy, and sensory sensitivity allowed the identification of three clusters of subjects, namely, a group in which ATs were highly prevalent (AT cluster), another in which such features were not present (no-AT cluster), and a subset of participants showing an intermediate pattern of AT expression (intermediate cluster). Of the aforementioned clinical and psychopathological features, the AT prevalence was the one driving the identification of the clusters.

Regarding sex distribution within clusters, and particularly the higher prevalence of female subjects in the no-AT cluster, our findings reflect the psychometric properties of the AQ and RAADS questionnaires. The AQ was originally validated in a predominantly male sample [31], and though a potential specific female presentation of ASDs has emerged in literature [32–35], most psychometric scales for assessing ASDs are constructed on the classical phenotype of the disorder, thereby being more accurate in identifying male patients with ASDs [36–40]. Even though the prevalence of male subjects was higher in the intermediate cluster compared to the AT cluster, male subjects still comprise almost 50% of the intermediate cluster. This is even more relevant considering the overall female predominance of the total sample. The higher prevalence of nonbinary subjects in the AT cluster is consistent with existing literature on the distribution of ATs in both general and clinical populations [41]. For instance, another study has reported that transgender and gender-diverse individuals scored significantly higher on self-report measures of ATs compared to cisgender individuals [42].

In the present study, individuals with a higher prevalence of ATs displayed overall greater symptomatology across various psychopathological dimensions, including mood, anxiety, severity of EDs, psychotic symptoms, and personality structure. This is in line with recent literature about the relationship between ATs and other psychiatric disorders [43, 44].

In our sample, there was not a statistically significant difference between the clusters with regard to the number of subjects scoring above the cutoff at BDI-II and STAI-Y1/Y2 scales. Nonetheless, subjects in the intermediate and AT clusters exhibited statistically significant higher scores on both scales, thus indicating a higher level of overall affective symptomatology. Previous literature has already highlighted the relationship between ATs and affective disorders, namely, depression, either bipolar or unipolar [45–48], and anxiety disorders [19, 49]. Moreover, studies have focused on the striking clinical similarities between ATs and social anxiety disorder (SAD) manifestations, such as social avoidance, difficulties being in public, and avoidance of eye contact [50–52]. These shared features can sometimes make it difficult to adequately distinguish the two conditions in clinical practice [53], also considering the well-researched frequent comorbidity between full-blown ASDs and SAD [54–57].

The same trend was observed for the scales assessing EDs symptoms and psychosis risk, wherein participants with higher ATs obtained higher scores compared to those in the intermediate and no-AT clusters. There is now a substantial body of literature highlighting the shared psychopathological features between ASDs and EDs (especially, but not exclusively, anorexia nervosa, AN). These include atypical social cognition, difficulties in processing emotions, cognitive rigidity [58], and an impaired theory of mind [59]. Furthermore, research indicates a higher prevalence of ATs in both AN [60–62] and other EDs [12, 63].

As for psychotic spectrum disorders and ASDs, an increasing number of papers have pointed out the similarities in the clinical presentation of these conditions, as well as the higher prevalence of ATs in patients diagnosed with schizophrenia spectrum disorders (SSDs) and the comorbidity between these syndromes [64, 65]. Social withdrawal, theory of mind deficits, and cognitive dysfunctions represent shared symptomatologic features between these conditions, and a conspicuous number of studies have identified genetic, biological, and familial overlap between SSDs and ASDs [66–69]. Furthermore, an increased occurrence of schizophrenia has been observed in individuals with ASDs compared to controls [70, 71], and this is also true for subthreshold ASD conditions, which show high prevalence rates in psychotic populations [72].

The higher scores obtained by AT subjects at the *Detachment* and *Disinhibition* domains of the PID-5-BF questionnaire reflect the clinical similarity between some features of ASDs (and ATs) and the anxious-avoidant psychopathological dimension, as noted in prior studies [73, 74]. Additionally, disinhibition is a commonly observed symptom in full-blown ASDs, and it is worth mentioning that ASDs are often comorbid with obsessive-compulsive disorder (OCD) and impulse control disorders [75–77], which have all been classified among the so-called obsessive-compulsive spectrum disorders [78]. On the other hand, the higher scores observed in the *Negative Affect* and *Psychoticism* domains mirror the aforementioned considerations about BDI-II, STAY, and PQ-16 results.

Regarding narcissistic traits, in our study participants with more ATs displayed a higher amount of narcissistic vulnerability. It is now recognized that individuals with vulnerable narcissism face an increased risk of developing mental health conditions. Specifically, the dimensions of hiding and underestimating the self seem to be associated with suicidal ideation and a high frequency of non-suicidal self-harm [79]. The presence of greater vulnerable narcissism is also related to social avoidance, anxiety, and internalized depressive manifestations (low self-esteem and shame) [80, 81]. In line with these findings, our cohort consisted of young individuals seeking psychiatric assistance for various psychopathological concerns. Furthermore, it has been shown that a high prevalence of vulnerable, but not grandiose, narcissistic traits can be found among ASD patients [82], which recalls the definition of “naïve egocentrism” first postulated by Frith and colleagues referring to a certain self-absorption intrinsic to ASDs [83].

Lastly, the results of our discrimination analysis hint at the possibility that ATs may predispose to an overall greater psychopathological burden. This is evidenced by the accurate separation of AT clusters by the scores obtained at the administered psychometric scales.

The findings of this study must be interpreted in light of the following limitations. First, reliance on self-report scales for data collection may introduce biases due to subjective perceptions and social desirability biases. Second, the available ASDs screening scales might not adequately capture the female phenotype of ASDs, possibly resulting in the oversight of female participants exhibiting ATs. Additionally, the absence of a control group limits the ability to compare the observed findings with those of individuals without psychopathological conditions. Third, we did not include in our analysis important variables such as family history and substance use, since they were not consistently disclosed by every subject during the first consultation; moreover, further statistical analyses such as a stratification of the results based on demographic variables (e.g., living environment) were not feasible due to the unequal distribution of participants across the living environment categories (i.e., most subjects lived with their family). Lastly, the cross-sectional nature of the study restricts the possibility of establishing causal relationships or tracking longitudinal changes in psychopathological symptoms over time.

In conclusion, this study highlights the high prevalence of ATs in a group of young individuals struggling with mental health concerns. Additionally, subjects exhibiting more ATs displayed an overall greater severity of psychiatric symptoms across various psychopathological domains, including mood, anxiety, EDs, psychotic symptoms, and personality structure. The findings also underscore the necessity of adopting a dimensional approach to psychopathology to better understand the complex interplay of symptoms and facilitate tailored interventions. Future studies with a larger sample, a longitudinal nature, and a control group may provide further insights into the relationship between ATs and other psychiatric disorders.

## Data Availability

The data that support the findings of this study are available on request from the corresponding author.
